# Supplementary material for: Temporal predictability does not impact attentional blink performance: effects of fixed vs. random inter-trial intervals
Source: PeerJ. 2020 Mar 5;8:e8677. doi: 10.7717/peerj.8677 (PMC7060903; doi:10.7717/peerj.8677)
Supplement: Supplemental Information 3 [file peerj-08-8677-s003.jasp › index.html]

JASP 


# Results

## Bayesian Paired Samples T-Test

| Bayesian Paired Samples T-Test | | | | | | | | | |
| --- | --- | --- | --- | --- | --- | --- | --- | --- | --- |
|  | |  | |  | | BF₀₁ | | error % | |
| Random Interval Blink Magnitude |  | - |  | Fixed Interval Blink Magnitude |  | 4.612 |  | 0.007 |  |
| T1 incorrect, fixed |  | - |  | T1 incorrect, random |  | 4.252 |  | 0.004 |  |
|  | | | | | | | | | |

### Inferential Plots

#### Random Interval Blink Magnitude - Fixed Interval Blink Magnitude

##### Prior and Posterior

##### Bayes Factor Robustness Check

##### Sequential Analysis

#### T1 incorrect, fixed - T1 incorrect, random

##### Prior and Posterior

##### Bayes Factor Robustness Check

##### Sequential Analysis

| Descriptives | | | | | | | | | | | | | |
| --- | --- | --- | --- | --- | --- | --- | --- | --- | --- | --- | --- | --- | --- |
|  | | | | | | | | | | 95% Credible Interval | | | |
|  | | N | | Mean | | SD | | SE | | Lower | | Upper | |
| Random Interval Blink Magnitude |  | 30.000 |  | 0.167 |  | 0.164 |  | 0.030 |  | 0.106 |  | 0.229 |  |
| Fixed Interval Blink Magnitude |  | 30.000 |  | 0.182 |  | 0.145 |  | 0.026 |  | 0.128 |  | 0.236 |  |
| T1 incorrect, fixed |  | 30.000 |  | 0.118 |  | 0.078 |  | 0.014 |  | 0.089 |  | 0.147 |  |
| T1 incorrect, random |  | 30.000 |  | 0.134 |  | 0.104 |  | 0.019 |  | 0.095 |  | 0.173 |  |
|  | | | | | | | | | | | | | |

### Descriptives Plots

#### Random Interval Blink Magnitude - Fixed Interval Blink Magnitude

#### T1 incorrect, fixed - T1 incorrect, random
