# Supplementary figures and images for: Temporal predictability does not impact attentional blink performance: effects of fixed vs. random inter-trial intervals
Source: PeerJ. 2020 Mar 5;8:e8677. doi: 10.7717/peerj.8677 (PMC7060903; doi:10.7717/peerj.8677)

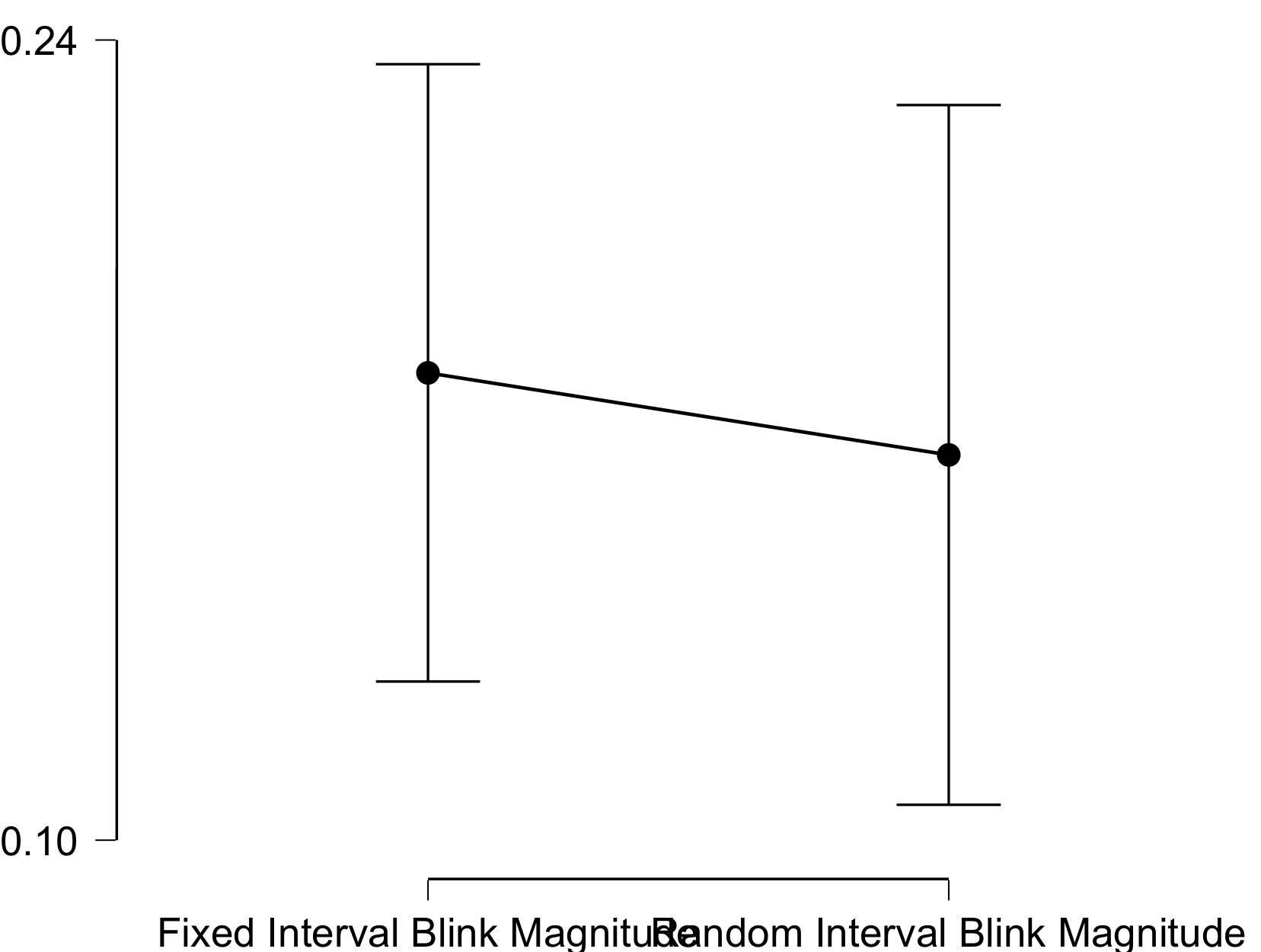

Supplement: Supplemental Information 3 [file peerj-08-8677-s003.jasp › resources/1/_8.png]

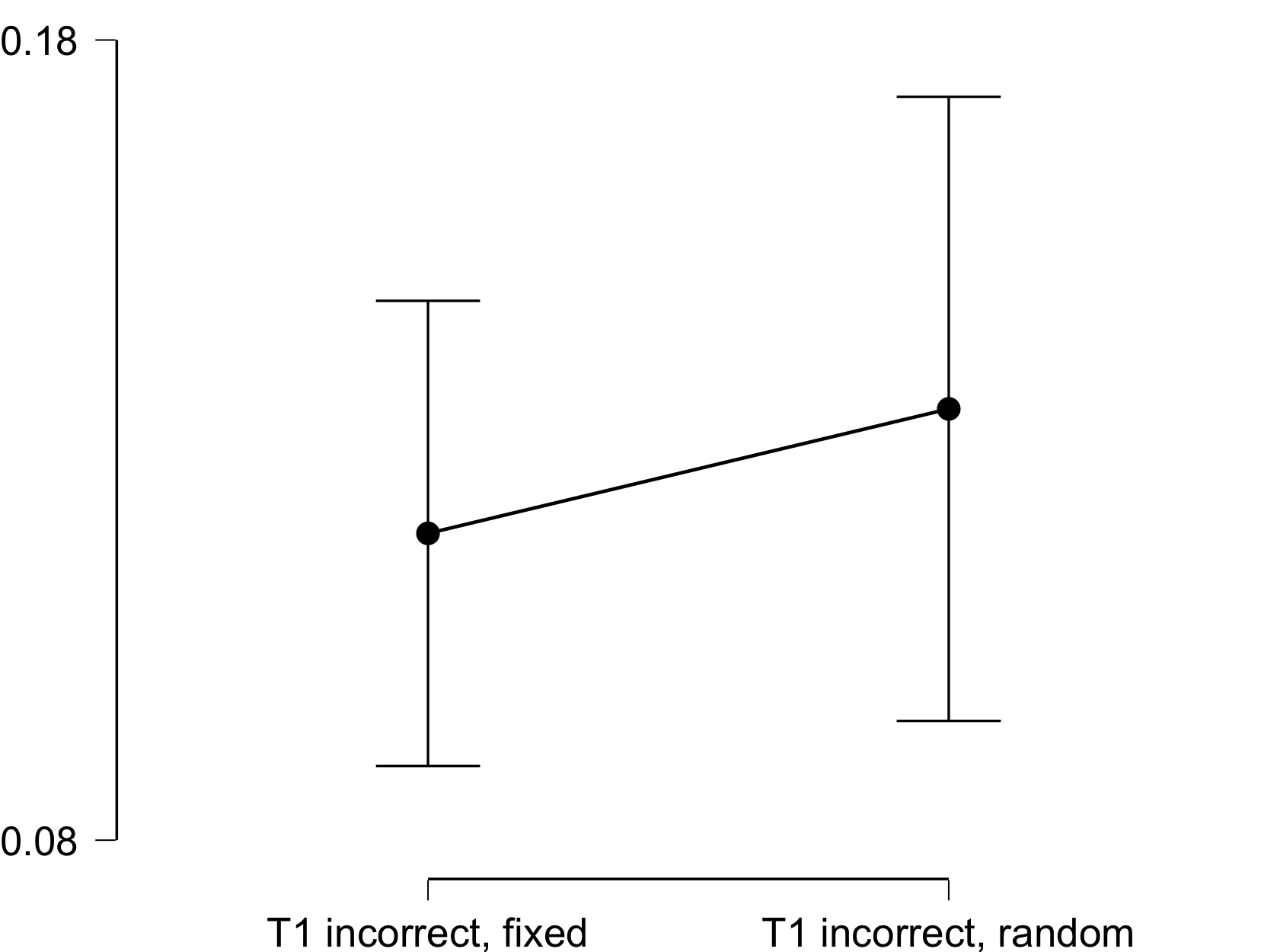

Supplement: Supplemental Information 3 [file peerj-08-8677-s003.jasp › resources/1/_9.png]

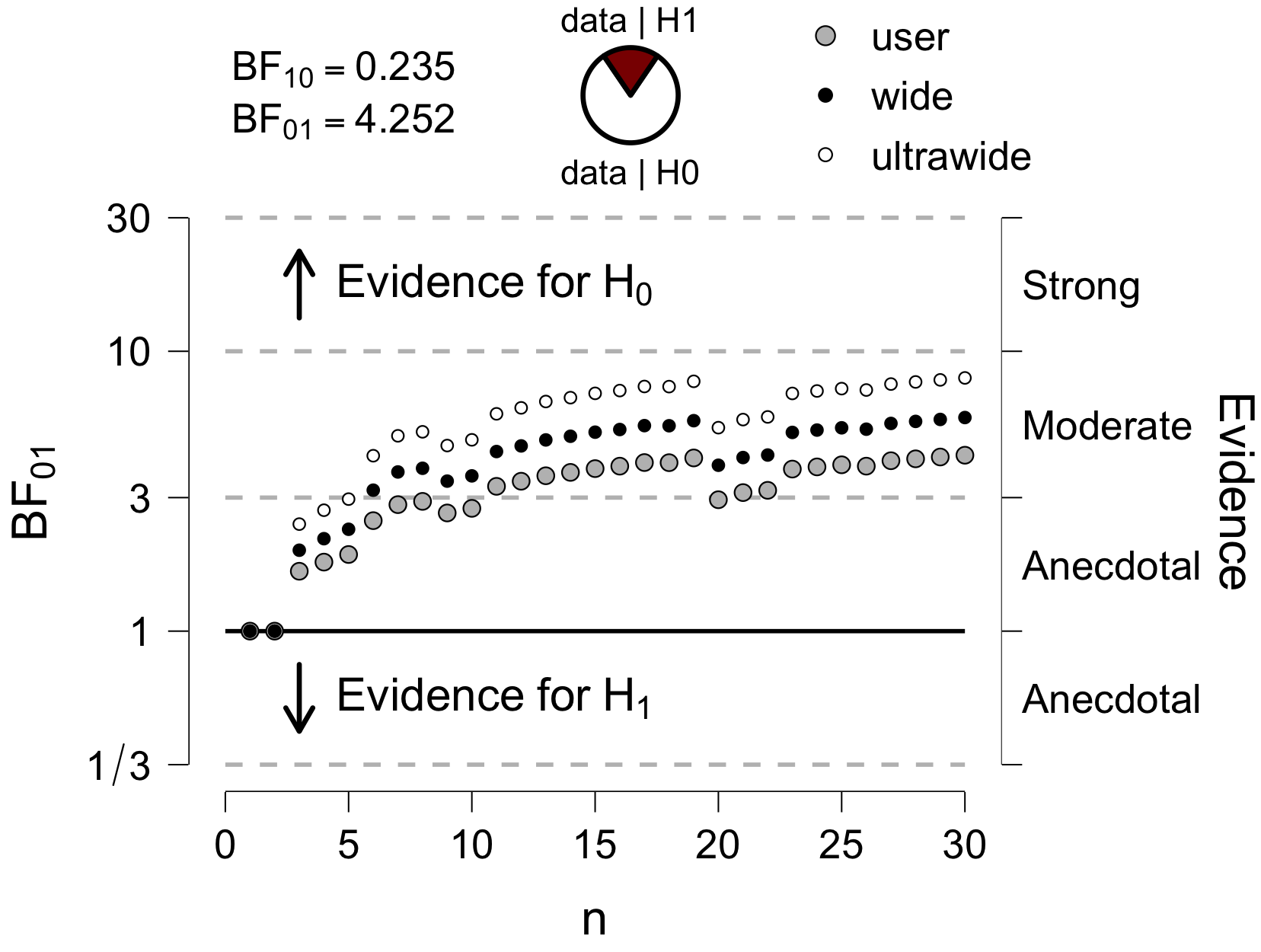

Supplement: Supplemental Information 3 [file peerj-08-8677-s003.jasp › resources/1/_15.png]

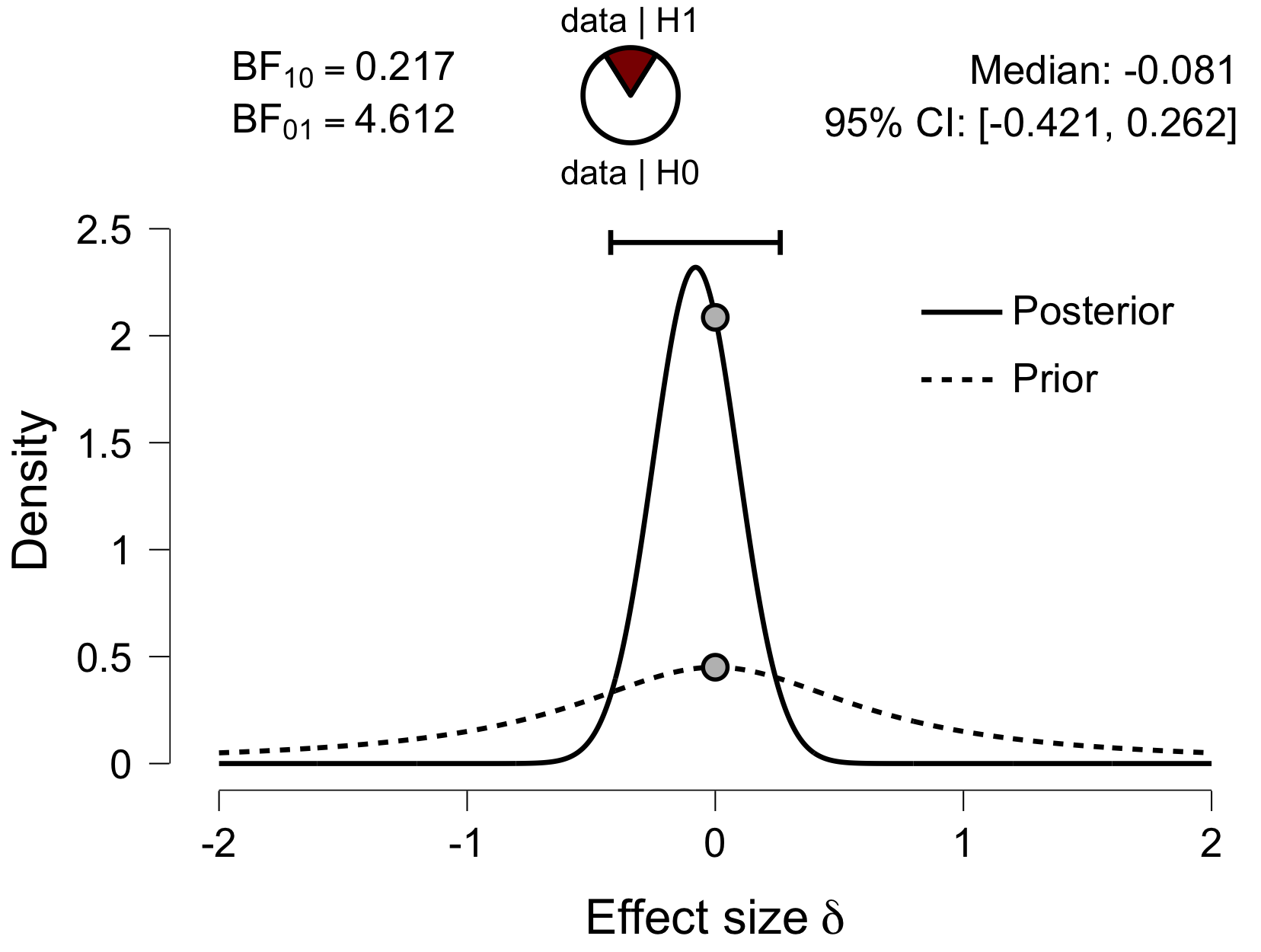

Supplement: Supplemental Information 3 [file peerj-08-8677-s003.jasp › resources/1/_2.png]

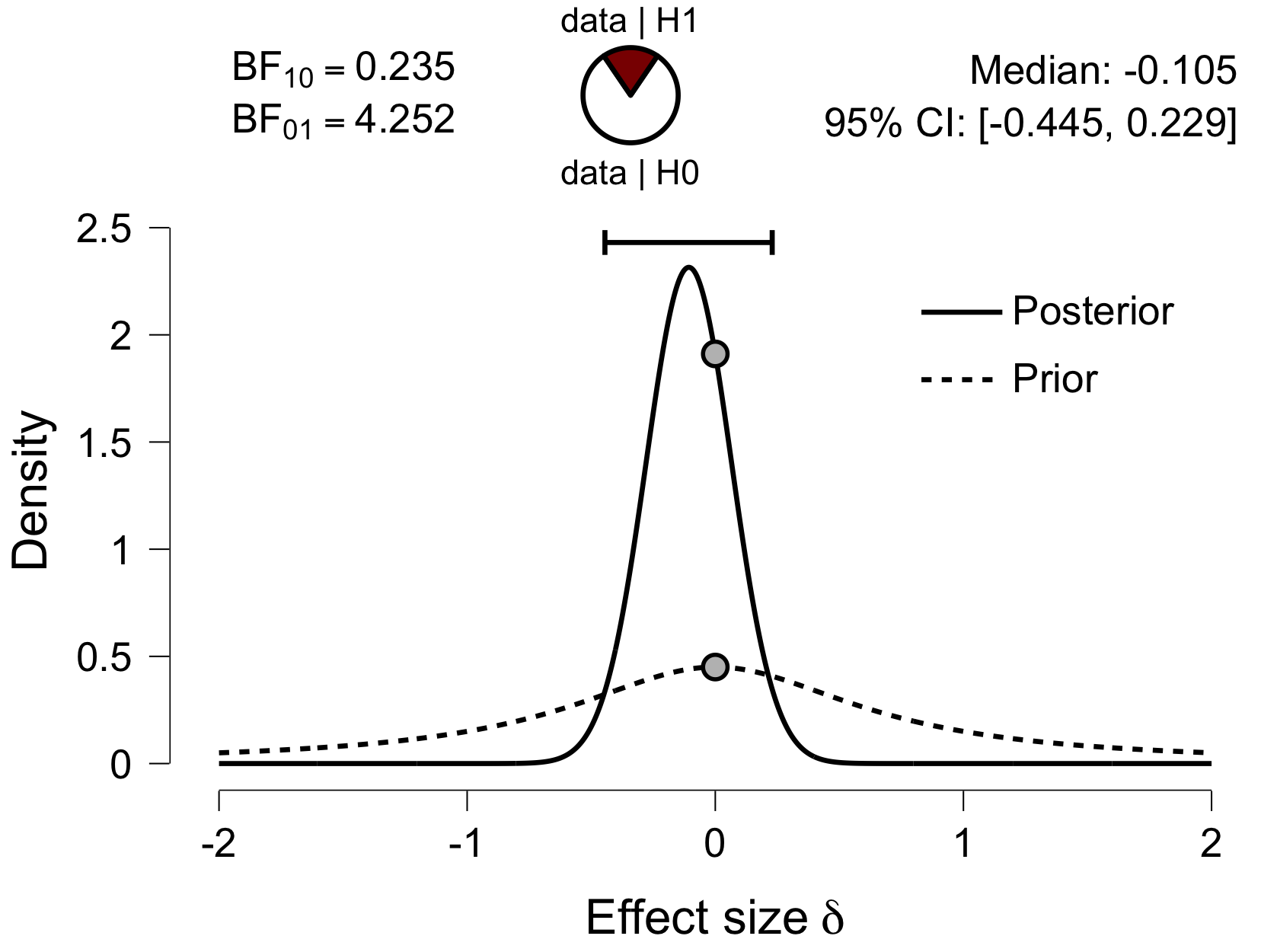

Supplement: Supplemental Information 3 [file peerj-08-8677-s003.jasp › resources/1/_3.png]

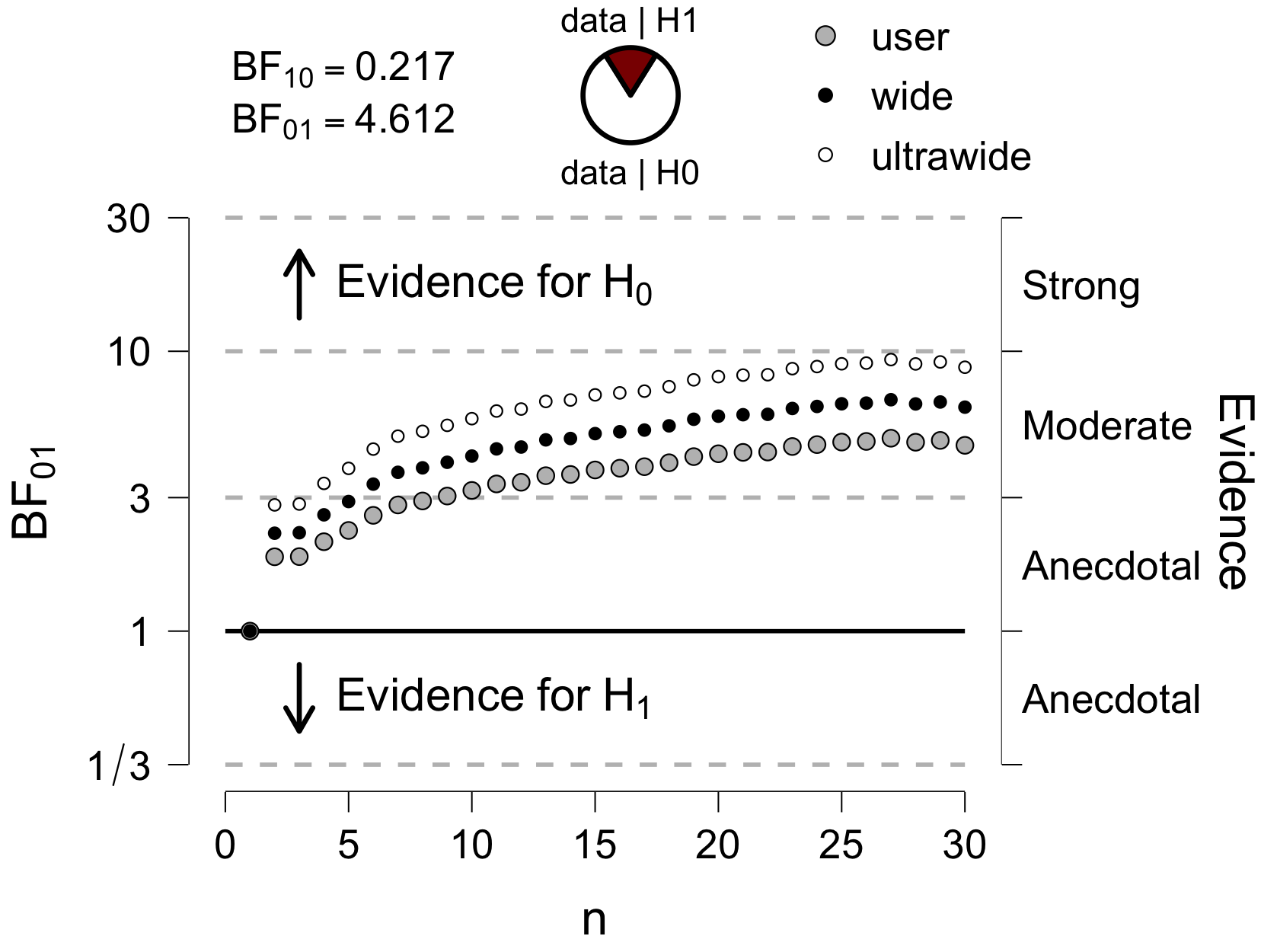

Supplement: Supplemental Information 3 [file peerj-08-8677-s003.jasp › resources/1/_14.png]

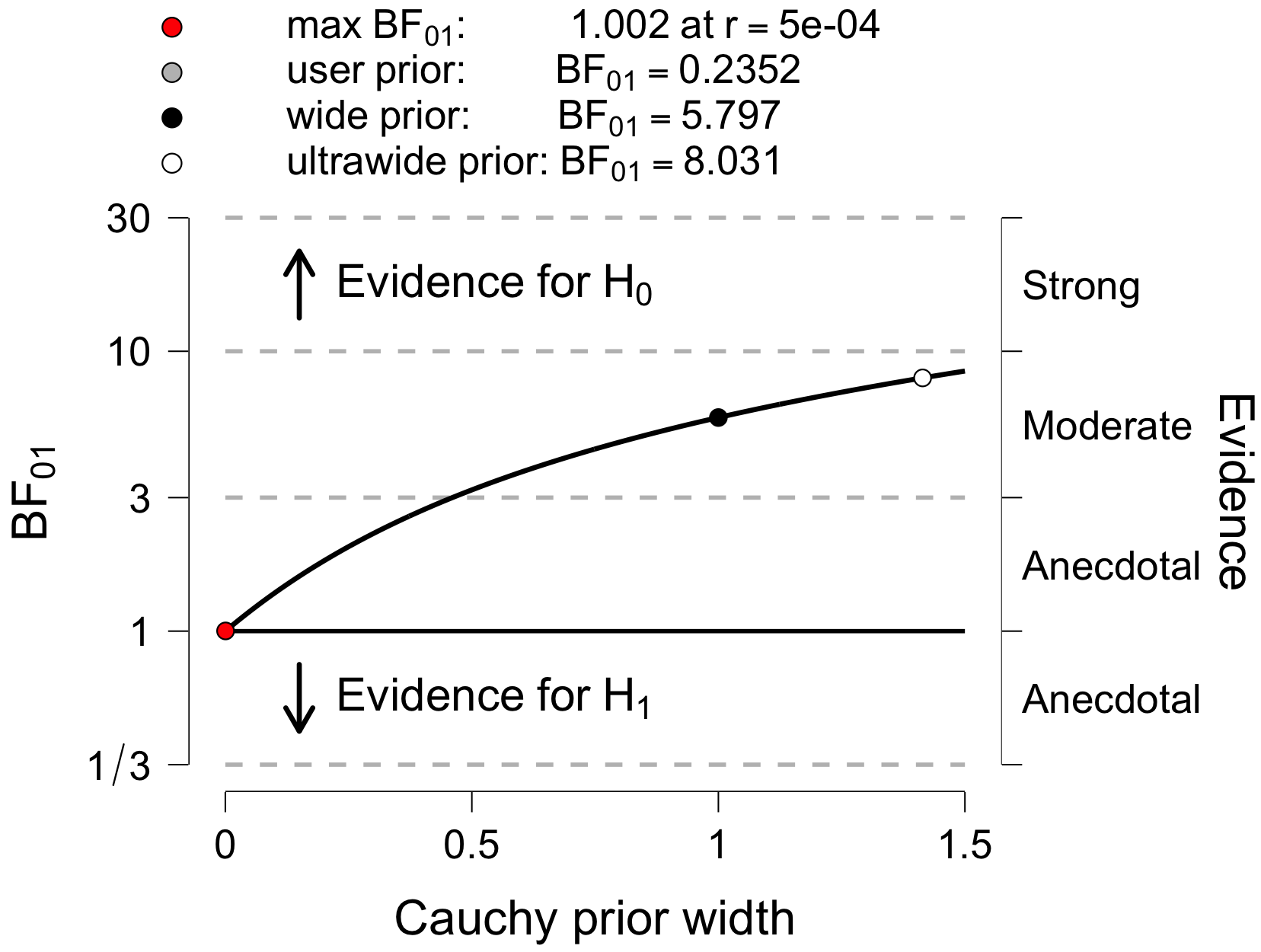

Supplement: Supplemental Information 3 [file peerj-08-8677-s003.jasp › resources/1/_13.png]

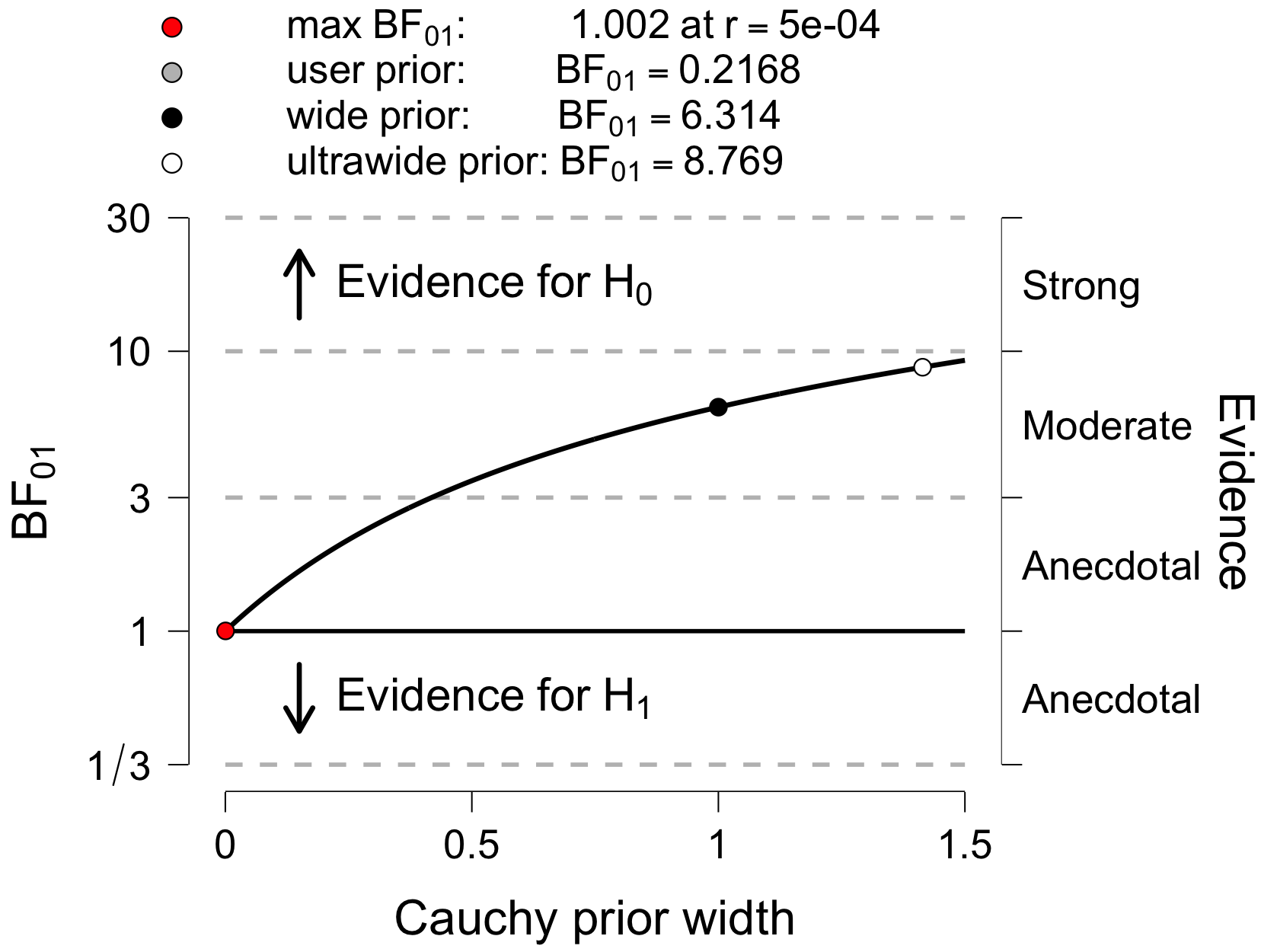

Supplement: Supplemental Information 3 [file peerj-08-8677-s003.jasp › resources/1/_4.png]

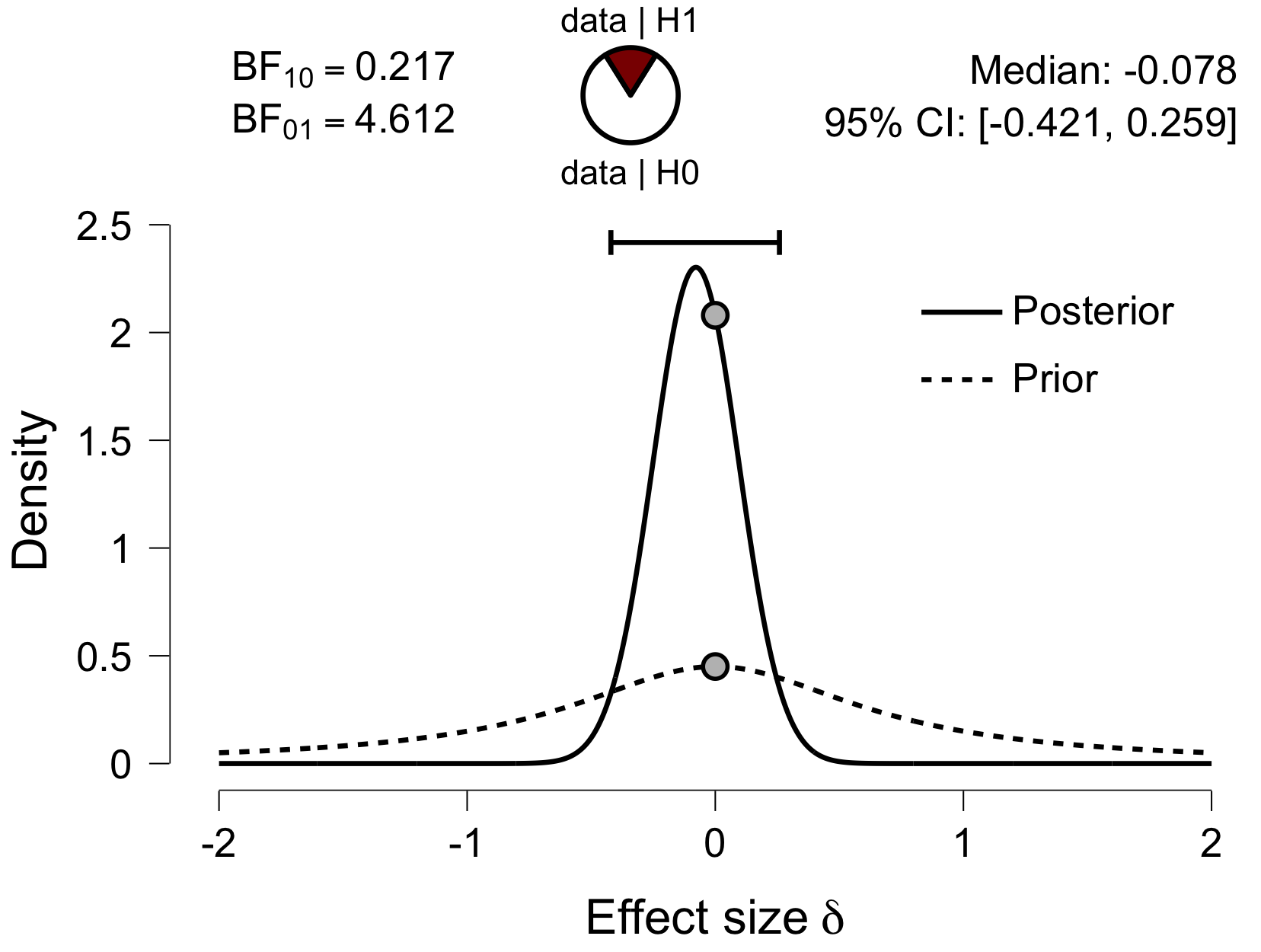

Supplement: Supplemental Information 3 [file peerj-08-8677-s003.jasp › resources/1/_10.png]

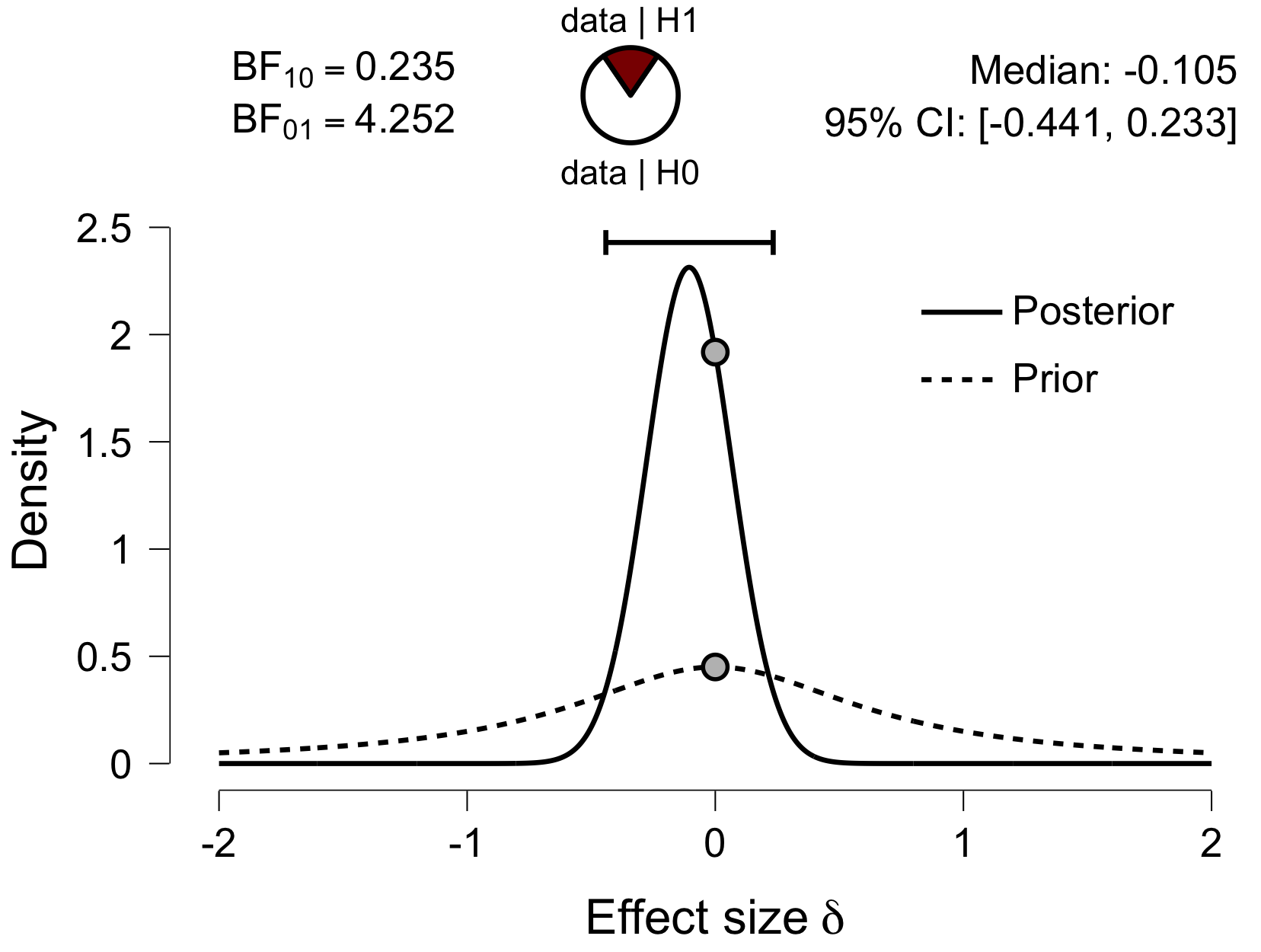

Supplement: Supplemental Information 3 [file peerj-08-8677-s003.jasp › resources/1/_11.png]

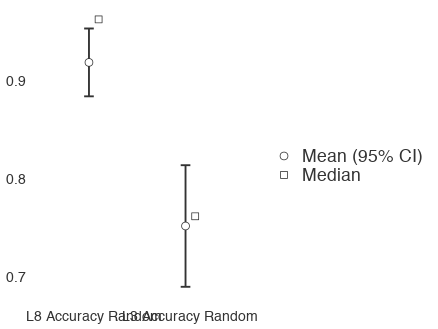

Supplement: Supplemental Information 5 [file peerj-08-8677-s005.omv › 02 ttestPS/resources/b38989597a06f3a1.png]

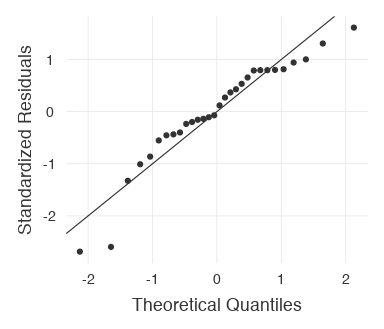

Supplement: Supplemental Information 5 [file peerj-08-8677-s005.omv › 02 ttestPS/resources/a537f09eeebf18ac.png]

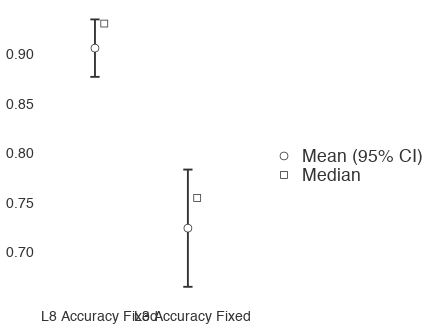

Supplement: Supplemental Information 5 [file peerj-08-8677-s005.omv › 02 ttestPS/resources/e6531fb68a5ec2b9.png]

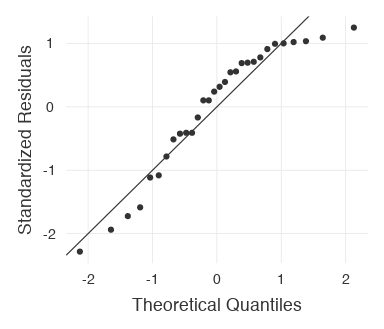

Supplement: Supplemental Information 5 [file peerj-08-8677-s005.omv › 02 ttestPS/resources/4ab04b0d596dd472.png]

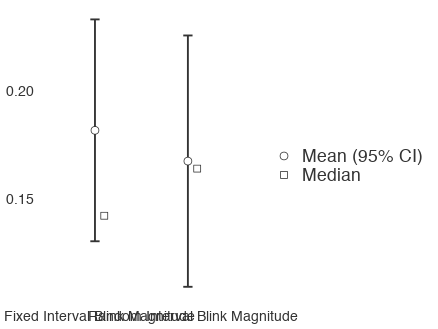

Supplement: Supplemental Information 5 [file peerj-08-8677-s005.omv › 02 ttestPS/resources/2a6b79799bc091e1.png]

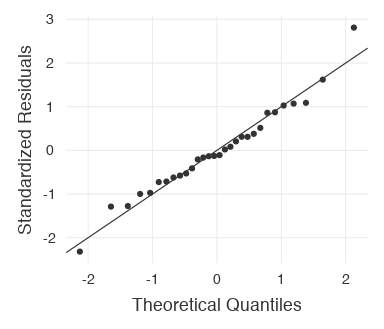

Supplement: Supplemental Information 5 [file peerj-08-8677-s005.omv › 02 ttestPS/resources/ec0564de4d0e85fb.png]

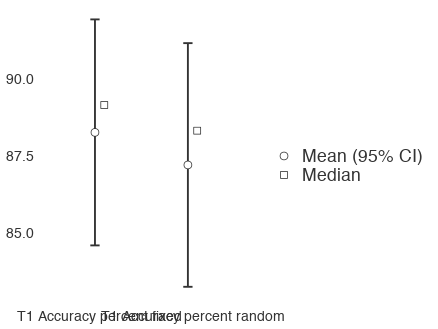

Supplement: Supplemental Information 5 [file peerj-08-8677-s005.omv › 02 ttestPS/resources/886cb58b20741ecc.png]

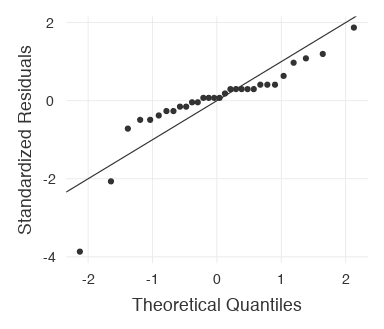

Supplement: Supplemental Information 5 [file peerj-08-8677-s005.omv › 02 ttestPS/resources/ae928d0ee5b68c4c.png]

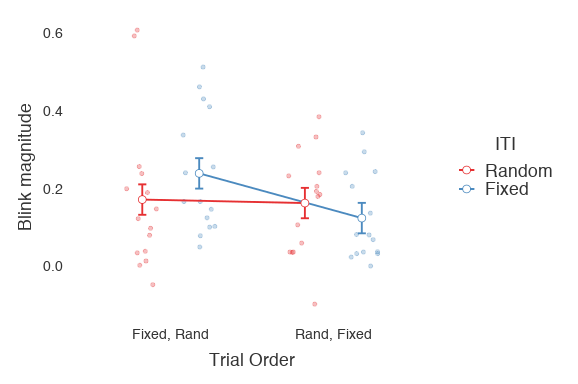

Supplement: Supplemental Information 5 [file peerj-08-8677-s005.omv › 04 anovaRM/resources/82a331860c462d80.png]

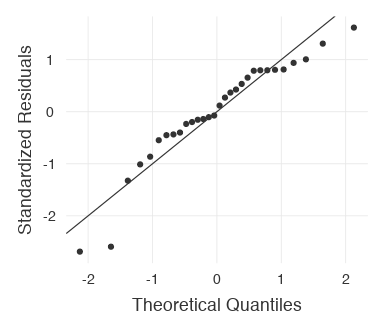

Supplement: Supplemental Information 7 — This analysis in jamovi shows that removing non-responses to T2 from all the analyses produces extremely similar results to those reported in the paper (where non-responses were scored as incorrect). [file peerj-08-8677-s007.omv › 02 ttestPS/resources/3d2d642b26d26d4d.png]

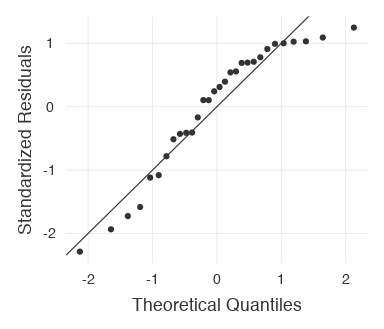

Supplement: Supplemental Information 7 — This analysis in jamovi shows that removing non-responses to T2 from all the analyses produces extremely similar results to those reported in the paper (where non-responses were scored as incorrect). [file peerj-08-8677-s007.omv › 02 ttestPS/resources/a0f591c0914b7271.png]

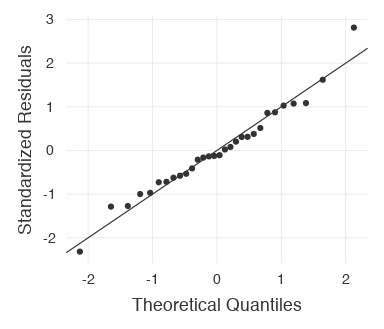

Supplement: Supplemental Information 7 — This analysis in jamovi shows that removing non-responses to T2 from all the analyses produces extremely similar results to those reported in the paper (where non-responses were scored as incorrect). [file peerj-08-8677-s007.omv › 02 ttestPS/resources/749df8678e079c7b.png]

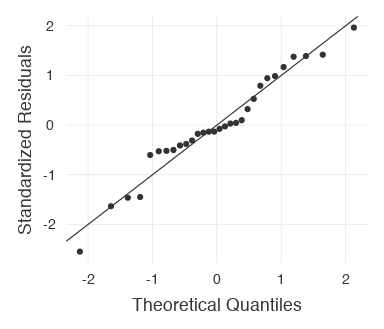

Supplement: Supplemental Information 7 — This analysis in jamovi shows that removing non-responses to T2 from all the analyses produces extremely similar results to those reported in the paper (where non-responses were scored as incorrect). [file peerj-08-8677-s007.omv › 02 ttestPS/resources/38f88e3c0e859629.png]

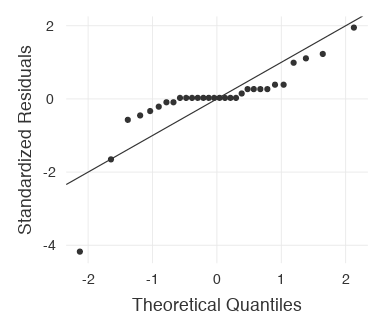

Supplement: Supplemental Information 7 — This analysis in jamovi shows that removing non-responses to T2 from all the analyses produces extremely similar results to those reported in the paper (where non-responses were scored as incorrect). [file peerj-08-8677-s007.omv › 02 ttestPS/resources/9eddc75691649ac8.png]

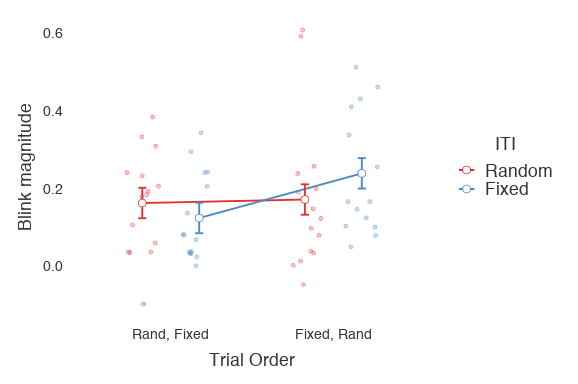

Supplement: Supplemental Information 7 — This analysis in jamovi shows that removing non-responses to T2 from all the analyses produces extremely similar results to those reported in the paper (where non-responses were scored as incorrect). [file peerj-08-8677-s007.omv › 03 anovaRM/resources/a838c0094424a5ef.png]

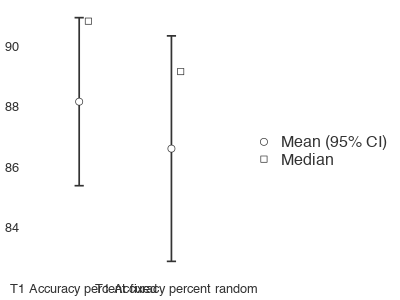

Supplement: Supplemental Information 8 — This file should enable the reader to replicate all the statistical analyses in the paper using any statistical program [file peerj-08-8677-s008.csv › 06 ttestPS/resources/28ec30ddae50b629.png]

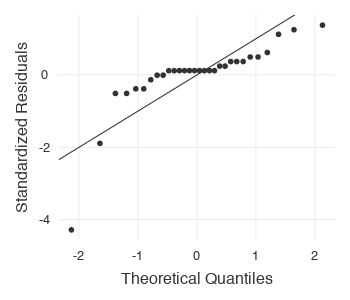

Supplement: Supplemental Information 8 — This file should enable the reader to replicate all the statistical analyses in the paper using any statistical program [file peerj-08-8677-s008.csv › 06 ttestPS/resources/ecddfb9796793fb8.png]

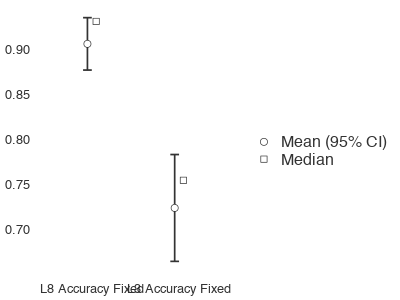

Supplement: Supplemental Information 8 — This file should enable the reader to replicate all the statistical analyses in the paper using any statistical program [file peerj-08-8677-s008.csv › 06 ttestPS/resources/194264ed292a579f.png]

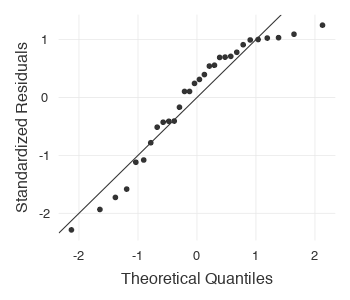

Supplement: Supplemental Information 8 — This file should enable the reader to replicate all the statistical analyses in the paper using any statistical program [file peerj-08-8677-s008.csv › 06 ttestPS/resources/0432d3de5c4c2ad0.png]

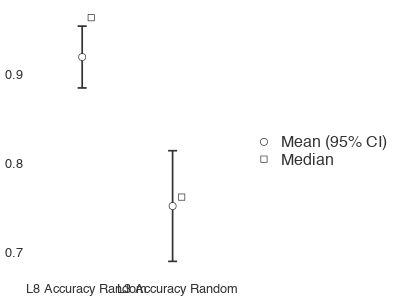

Supplement: Supplemental Information 8 — This file should enable the reader to replicate all the statistical analyses in the paper using any statistical program [file peerj-08-8677-s008.csv › 06 ttestPS/resources/d9a2630ca6cebce4.png]

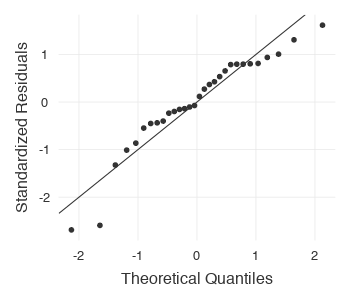

Supplement: Supplemental Information 8 — This file should enable the reader to replicate all the statistical analyses in the paper using any statistical program [file peerj-08-8677-s008.csv › 06 ttestPS/resources/c85fa4c6fd586a1d.png]

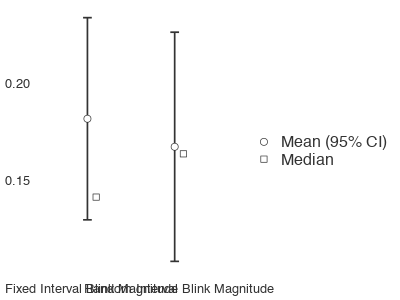

Supplement: Supplemental Information 8 — This file should enable the reader to replicate all the statistical analyses in the paper using any statistical program [file peerj-08-8677-s008.csv › 06 ttestPS/resources/d2b60db4811b176d.png]

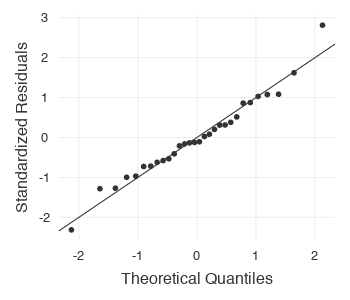

Supplement: Supplemental Information 8 — This file should enable the reader to replicate all the statistical analyses in the paper using any statistical program [file peerj-08-8677-s008.csv › 06 ttestPS/resources/65987e93f87c6539.png]

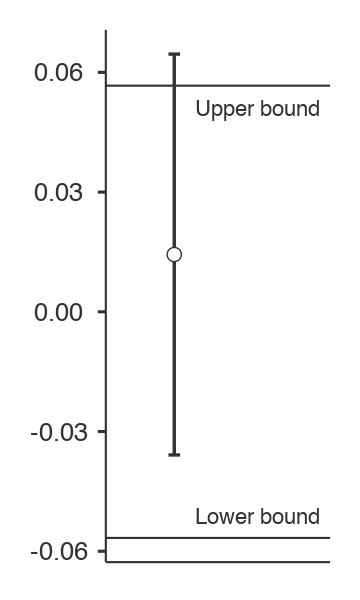

Supplement: Supplemental Information 8 — This file should enable the reader to replicate all the statistical analyses in the paper using any statistical program [file peerj-08-8677-s008.csv › 08 dataTOSTpaired/resources/3b191edee052a007.png]

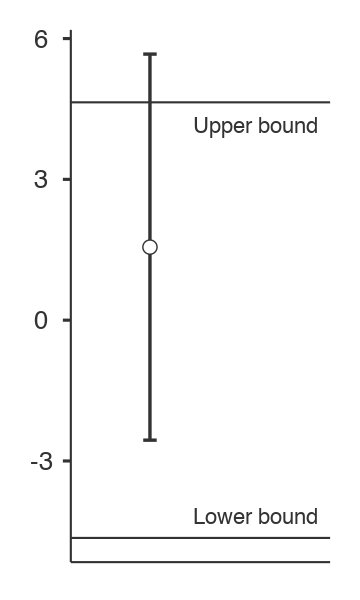

Supplement: Supplemental Information 8 — This file should enable the reader to replicate all the statistical analyses in the paper using any statistical program [file peerj-08-8677-s008.csv › 08 dataTOSTpaired/resources/32e769d796d738ef.png]
