# Supplementary material for: Temporal predictability does not impact attentional blink performance: effects of fixed vs. random inter-trial intervals
Source: PeerJ. 2020 Mar 5;8:e8677. doi: 10.7717/peerj.8677 (PMC7060903; doi:10.7717/peerj.8677)
Supplement: Supplemental Information 5 [file peerj-08-8677-s005.omv › index.html]

Results


# Paired Samples T-Test

| Paired Samples T-Test | | | | | | | | | | | | | | | | | | | | | | | |
| --- | --- | --- | --- | --- | --- | --- | --- | --- | --- | --- | --- | --- | --- | --- | --- | --- | --- | --- | --- | --- | --- | --- | --- |
|  | | | | | | | | | | | | | | | | | | 95% Confidence Interval | | | |  | |
|  | |  | |  | | statistic | | ±% | | df | | p | | Mean difference | | SE difference | | Lower | | Upper | | Cohen's d | |
| L8 Accuracy Random |  | L3 Accuracy Random |  | Student's t |  | 5.590 |  |  |  | 29.0 |  | < .001 |  | 0.1674 |  | 0.0299 |  | 0.1062 |  | 0.2286 |  | 1.0206 |  |
|  |  |  |  | Bayes factor₁₀ |  | 4044.943 |  | 8.35e-9 |  |  |  |  |  |  |  |  |  |  |  |  |  |  |  |
|  |  |  |  | Wilcoxon W |  | 446 |  |  |  |  | | < .001 |  | 0.1480 |  | 0.0299 |  | 0.1020 |  | 0.2115 |  | 1.0206 |  |
| L8 Accuracy Fixed |  | L3 Accuracy Fixed |  | Student's t |  | 6.885 |  |  |  | 29.0 |  | < .001 |  | 0.1817 |  | 0.0264 |  | 0.1277 |  | 0.2357 |  | 1.2569 |  |
|  |  |  |  | Bayes factor₁₀ |  | 105892.122 |  | 4.07e-10 |  |  |  |  |  |  |  |  |  |  |  |  |  |  |  |
|  |  |  |  | Wilcoxon W |  | 465 |  |  |  |  | | < .001 |  | 0.1675 |  | 0.0264 |  | 0.1130 |  | 0.2400 |  | 1.2569 |  |
| Fixed Interval Blink Magnitude |  | Random Interval Blink Magnitude |  | Student's t |  | 0.486 |  |  |  | 29.0 |  | 0.631 |  | 0.0144 |  | 0.0296 |  | -0.0461 |  | 0.0748 |  | 0.0887 |  |
|  |  |  |  | Bayes factor₁₀ |  | 0.217 |  | 7.35e-5 |  |  |  |  |  |  |  |  |  |  |  |  |  |  |  |
|  |  |  |  | Wilcoxon W |  | 268 |  |  |  |  | | 0.472 |  | 0.0225 |  | 0.0296 |  | -0.0389 |  | 0.0755 |  | 0.0887 |  |
| T1 Accuracy percent fixed |  | T1 Accuracy percent random |  | Student's t |  | 0.390 |  |  |  | 29.0 |  | 0.699 |  | 1.0556 |  | 2.7046 |  | -4.4759 |  | 6.5870 |  | 0.0713 |  |
|  |  |  |  | Bayes factor₁₀ |  | 0.209 |  | 9.25e-5 |  |  |  |  |  |  |  |  |  |  |  |  |  |  |  |
|  |  |  |  | Wilcoxon W |  | 169 | ᵃ |  |  |  | | 0.869 |  | -4.72e−4 |  | 2.7046 |  | -4.1670 |  | 3.3341 |  | 0.0713 |  |
|  |  |  |  |  |  |  |  |  |  |  |  |  |  |  |  |  |  |  |  |  |  |  |  |
| --- | --- | --- | --- | --- | --- | --- | --- | --- | --- | --- | --- | --- | --- | --- | --- | --- | --- | --- | --- | --- | --- | --- | --- |
| ᵃ 4 pair(s) of values were tied | | | | | | | | | | | | | | | | | | | | | | | |
|  | | | | | | | | | | | | | | | | | | | | | | | |
| [3] [4] | | | | | | | | | | | | | | | | | | | | | | | |

| Tests of Normality | | | | | | | | | |
| --- | --- | --- | --- | --- | --- | --- | --- | --- | --- |
|
|  | |  | |  | | statistic | | p | |
| L8 Accuracy Random |  | L3 Accuracy Random |  | Shapiro-Wilk |  | 0.915 |  | 0.020 |  |
|  | |  | | Kolmogorov-Smirnov |  | 0.1246 |  | 0.694 |  |
|  | |  | | Anderson-Darling |  | 0.744 |  | 0.047 |  |
| L8 Accuracy Fixed |  | L3 Accuracy Fixed |  | Shapiro-Wilk |  | 0.909 |  | 0.014 |  |
|  | |  | | Kolmogorov-Smirnov |  | 0.1406 |  | 0.594 |  |
|  | |  | | Anderson-Darling |  | 0.914 |  | 0.017 |  |
| Fixed Interval Blink Magnitude |  | Random Interval Blink Magnitude |  | Shapiro-Wilk |  | 0.974 |  | 0.663 |  |
|  | |  | | Kolmogorov-Smirnov |  | 0.0857 |  | 0.980 |  |
|  | |  | | Anderson-Darling |  | 0.285 |  | 0.604 |  |
| T1 Accuracy percent fixed |  | T1 Accuracy percent random |  | Shapiro-Wilk |  | 0.794 |  | < .001 |  |
|  | |  | | Kolmogorov-Smirnov |  | 0.2116 |  | 0.136 |  |
|  | |  | | Anderson-Darling |  | 1.971 |  | < .001 |  |
|  |  |  |  |  |  |  |  |  |  |
| --- | --- | --- | --- | --- | --- | --- | --- | --- | --- |
|  | | | | | | | | | |
|  | | | | | | | | | |

| Descriptives | | | | | | | | | | | |
| --- | --- | --- | --- | --- | --- | --- | --- | --- | --- | --- | --- |
|
|  | | N | | Mean | | Median | | SD | | SE | |
| L8 Accuracy Random |  | 30 |  | 0.919 |  | 0.964 |  | 0.0970 |  | 0.0177 |  |
| L3 Accuracy Random |  | 30 |  | 0.752 |  | 0.762 |  | 0.1738 |  | 0.0317 |  |
| L8 Accuracy Fixed |  | 30 |  | 0.906 |  | 0.931 |  | 0.0811 |  | 0.0148 |  |
| L3 Accuracy Fixed |  | 30 |  | 0.724 |  | 0.755 |  | 0.1653 |  | 0.0302 |  |
| Fixed Interval Blink Magnitude |  | 30 |  | 0.182 |  | 0.142 |  | 0.1447 |  | 0.0264 |  |
| Random Interval Blink Magnitude |  | 30 |  | 0.168 |  | 0.164 |  | 0.1639 |  | 0.0299 |  |
| T1 Accuracy percent fixed |  | 30 |  | 88.278 |  | 89.166 |  | 10.2411 |  | 1.8698 |  |
| T1 Accuracy percent random |  | 30 |  | 87.222 |  | 88.333 |  | 11.0409 |  | 2.0158 |  |
|  |  |  |  |  |  |  |  |  |  |  |  |
| --- | --- | --- | --- | --- | --- | --- | --- | --- | --- | --- | --- |
|  | | | | | | | | | | | |
|  | | | | | | | | | | | |

## Plots

### L8 Accuracy Random - L3 Accuracy Random

#### 

#### 

### L8 Accuracy Fixed - L3 Accuracy Fixed

#### 

#### 

### Fixed Interval Blink Magnitude - Random Interval Blink Magnitude

#### 

#### 

### T1 Accuracy percent fixed - T1 Accuracy percent random

#### 

#### 

# Repeated Measures ANOVA

| Within Subjects Effects | | | | | | | | | | | | | |
| --- | --- | --- | --- | --- | --- | --- | --- | --- | --- | --- | --- | --- | --- |
|
|  | | Sum of Squares | | df | | Mean Square | | F | | p | | η²p | |
| ITI |  | 0.00310 |  | 1 |  | 0.00310 |  | 0.256 |  | 0.617 |  | 0.009 |  |
| ITI ✻ Trial Order |  | 0.04219 |  | 1 |  | 0.04219 |  | 3.493 |  | 0.072 |  | 0.111 |  |
| Residual |  | 0.33819 |  | 28 |  | 0.01208 |  |  |  |  |  |  |  |
|  |  |  |  |  |  |  |  |  |  |  |  |  |  |
| --- | --- | --- | --- | --- | --- | --- | --- | --- | --- | --- | --- | --- | --- |
| Note. Type 3 Sums of Squares | | | | | | | | | | | | | |
|  | | | | | | | | | | | | | |
| [5] | | | | | | | | | | | | | |

| Between Subjects Effects | | | | | | | | | | | | | |
| --- | --- | --- | --- | --- | --- | --- | --- | --- | --- | --- | --- | --- | --- |
|
|  | | Sum of Squares | | df | | Mean Square | | F | | p | | η²p | |
| Trial Order |  | 0.0577 |  | 1 |  | 0.0577 |  | 1.70 |  | 0.202 |  | 0.057 |  |
| Residual |  | 0.9480 |  | 28 |  | 0.0339 |  |  |  |  |  |  |  |
|  |  |  |  |  |  |  |  |  |  |  |  |  |  |
| --- | --- | --- | --- | --- | --- | --- | --- | --- | --- | --- | --- | --- | --- |
| Note. Type 3 Sums of Squares | | | | | | | | | | | | | |
|  | | | | | | | | | | | | | |
|  | | | | | | | | | | | | | |

## Estimated Marginal Means

### Trial Order ✻ ITI

#### 

[6]

# References

[1]
The jamovi project (2019). *jamovi*. (Version 1.1) [Computer Software]. Retrieved from https://www.jamovi.org.

[2]
R Core Team (2018). *R: A Language and envionment for statistical computing*. [Computer software]. Retrieved from https://cran.r-project.org/.

[3]
Morey, R. D., & Rouder, J. N. (2018). *BayesFactor: Computation of Bayes Factors for Common Designs*. [R package]. Retrieved from https://cran.r-project.org/package=BayesFactor.

[4]
Rouder, J. N., Speckman, P. L., Sun, D., Morey, R. D., & Iverson, G. (2009). Bayesian t tests for accepting and rejecting the null hypothesis. *Psychonomic Bulletin & Review, 16*, 225-237.

[5]
Singmann, H. (2018). *afex: Analysis of Factorial Experiments*. [R package]. Retrieved from https://cran.r-project.org/package=afex.

[6]
Lenth, R. (2018). *emmeans: Estimated Marginal Means, aka Least-Squares Means*. [R package]. Retrieved from https://cran.r-project.org/package=emmeans.
