# Supplementary material for: Temporal predictability does not impact attentional blink performance: effects of fixed vs. random inter-trial intervals
Source: PeerJ. 2020 Mar 5;8:e8677. doi: 10.7717/peerj.8677 (PMC7060903; doi:10.7717/peerj.8677)
Supplement: Supplemental Information 7 — This analysis in jamovi shows that removing non-responses to T2 from all the analyses produces extremely similar results to those reported in the paper (where non-responses were scored as incorrect). [file peerj-08-8677-s007.omv › index.html]

Results


# Paired Samples T-Test

| Paired Samples T-Test | | | | | | | | | | | | | | | | | | | | | | | |
| --- | --- | --- | --- | --- | --- | --- | --- | --- | --- | --- | --- | --- | --- | --- | --- | --- | --- | --- | --- | --- | --- | --- | --- |
|  | | | | | | | | | | | | | | | | | | 95% Confidence Interval | | | |  | |
|  | |  | |  | | statistic | | ±% | | df | | p | | Mean difference | | SE difference | | Lower | | Upper | | Cohen's d | |
| Lag8\_PropCorrect\_Rand |  | Lag3\_PropCorrect\_Rand |  | Student's t |  | 5.593 |  |  |  | 29.0 |  | < .001 |  | 0.16741 |  | 0.0299 |  | 0.1062 |  | 0.2286 |  | 1.0212 |  |
|  |  |  |  | Bayes factor₁₀ |  | 4076.439 |  | 8.31e-9 |  |  |  |  |  |  |  |  |  |  |  |  |  |  |  |
|  |  |  |  | Wilcoxon W |  | 446.0 |  |  |  |  | | < .001 |  | 0.1478 |  | 0.0299 |  | 0.1033 |  | 0.2115 |  | 1.0212 |  |
| Lag8\_PropCorrect\_Fixed |  | Lag3\_PropCorrect\_Fixed |  | Student's t |  | 6.884 |  |  |  | 29.0 |  | < .001 |  | 0.18177 |  | 0.0264 |  | 0.1278 |  | 0.2358 |  | 1.2569 |  |
|  |  |  |  | Bayes factor₁₀ |  | 105806.626 |  | 4.07e-10 |  |  |  |  |  |  |  |  |  |  |  |  |  |  |  |
|  |  |  |  | Wilcoxon W |  | 465.0 |  |  |  |  | | < .001 |  | 0.1675 |  | 0.0264 |  | 0.1128 |  | 0.2401 |  | 1.2569 |  |
| BlinkMagnitude\_Fixed |  | BlinkMagnitude\_Rand |  | Student's t |  | 0.486 |  |  |  | 29.0 |  | 0.631 |  | 0.01435 |  | 0.0296 |  | -0.0461 |  | 0.0748 |  | 0.0886 |  |
|  |  |  |  | Bayes factor₁₀ |  | 0.217 |  | 7.36e-5 |  |  |  |  |  |  |  |  |  |  |  |  |  |  |  |
|  |  |  |  | Wilcoxon W |  | 267.0 |  |  |  |  | | 0.490 |  | 0.0222 |  | 0.0296 |  | -0.0382 |  | 0.0749 |  | 0.0886 |  |
| MedianRT\_Rand |  | MedianRT\_Fixed |  | Student's t |  | 0.295 |  |  |  | 29.0 |  | 0.770 |  | 0.00755 |  | 0.0255 |  | -0.0447 |  | 0.0598 |  | 0.0539 |  |
|  |  |  |  | Bayes factor₁₀ |  | 0.202 |  | 1.09e-4 |  |  |  |  |  |  |  |  |  |  |  |  |  |  |  |
|  |  |  |  | Wilcoxon W |  | 255.0 |  |  |  |  | | 0.655 |  | 0.0127 |  | 0.0255 |  | -0.0518 |  | 0.0519 |  | 0.0539 |  |
| T1 accuracy fixed |  | T1 Accuracy Random |  | Student's t |  | 0.153 |  |  |  | 29.0 |  | 0.879 |  | 0.00389 |  | 0.0253 |  | -0.0479 |  | 0.0557 |  | 0.0280 |  |
|  |  |  |  | Bayes factor₁₀ |  | 0.197 |  | 1.26e-4 |  |  |  |  |  |  |  |  |  |  |  |  |  |  |  |
|  |  |  |  | Wilcoxon W |  | 82.0 | ᵃ |  |  |  | | 0.614 |  | -0.0124 |  | 0.0253 |  | -0.0750 |  | 0.0417 |  | 0.0280 |  |
|  |  |  |  |  |  |  |  |  |  |  |  |  |  |  |  |  |  |  |  |  |  |  |  |
| --- | --- | --- | --- | --- | --- | --- | --- | --- | --- | --- | --- | --- | --- | --- | --- | --- | --- | --- | --- | --- | --- | --- | --- |
| ᵃ 11 pair(s) of values were tied | | | | | | | | | | | | | | | | | | | | | | | |
|  | | | | | | | | | | | | | | | | | | | | | | | |
| [3] [4] | | | | | | | | | | | | | | | | | | | | | | | |

| Tests of Normality | | | | | | | | | |
| --- | --- | --- | --- | --- | --- | --- | --- | --- | --- |
|
|  | |  | |  | | statistic | | p | |
| Lag8\_PropCorrect\_Rand |  | Lag3\_PropCorrect\_Rand |  | Shapiro-Wilk |  | 0.915 |  | 0.020 |  |
|  | |  | | Kolmogorov-Smirnov |  | 0.1259 |  | 0.682 |  |
|  | |  | | Anderson-Darling |  | 0.744 |  | 0.047 |  |
| Lag8\_PropCorrect\_Fixed |  | Lag3\_PropCorrect\_Fixed |  | Shapiro-Wilk |  | 0.909 |  | 0.014 |  |
|  | |  | | Kolmogorov-Smirnov |  | 0.1416 |  | 0.585 |  |
|  | |  | | Anderson-Darling |  | 0.914 |  | 0.017 |  |
| BlinkMagnitude\_Fixed |  | BlinkMagnitude\_Rand |  | Shapiro-Wilk |  | 0.974 |  | 0.657 |  |
|  | |  | | Kolmogorov-Smirnov |  | 0.0858 |  | 0.966 |  |
|  | |  | | Anderson-Darling |  | 0.286 |  | 0.602 |  |
| MedianRT\_Rand |  | MedianRT\_Fixed |  | Shapiro-Wilk |  | 0.962 |  | 0.351 |  |
|  | |  | | Kolmogorov-Smirnov |  | 0.1396 |  | 0.556 |  |
|  | |  | | Anderson-Darling |  | 0.530 |  | 0.162 |  |
| T1 accuracy fixed |  | T1 Accuracy Random |  | Shapiro-Wilk |  | 0.709 |  | < .001 |  |
|  | |  | | Kolmogorov-Smirnov |  | 0.2633 |  | 0.031 |  |
|  | |  | | Anderson-Darling |  | 3.099 |  | < .001 |  |
|  |  |  |  |  |  |  |  |  |  |
| --- | --- | --- | --- | --- | --- | --- | --- | --- | --- |
|  | | | | | | | | | |
|  | | | | | | | | | |

## Plots

### Lag8\_PropCorrect\_Rand - Lag3\_PropCorrect\_Rand

#### 

### Lag8\_PropCorrect\_Fixed - Lag3\_PropCorrect\_Fixed

#### 

### BlinkMagnitude\_Fixed - BlinkMagnitude\_Rand

#### 

### MedianRT\_Rand - MedianRT\_Fixed

#### 

### T1 accuracy fixed - T1 Accuracy Random

#### 

# Repeated Measures ANOVA

| Within Subjects Effects | | | | | | | | | | | | | |
| --- | --- | --- | --- | --- | --- | --- | --- | --- | --- | --- | --- | --- | --- |
|
|  | | Sum of Squares | | df | | Mean Square | | F | | p | | η²p | |
| ITI |  | 0.00309 |  | 1 |  | 0.00309 |  | 0.256 |  | 0.617 |  | 0.009 |  |
| ITI ✻ Trial Order |  | 0.04216 |  | 1 |  | 0.04216 |  | 3.492 |  | 0.072 |  | 0.111 |  |
| Residual |  | 0.33807 |  | 28 |  | 0.01207 |  |  |  |  |  |  |  |
|  |  |  |  |  |  |  |  |  |  |  |  |  |  |
| --- | --- | --- | --- | --- | --- | --- | --- | --- | --- | --- | --- | --- | --- |
| Note. Type 3 Sums of Squares | | | | | | | | | | | | | |
|  | | | | | | | | | | | | | |
| [5] | | | | | | | | | | | | | |

| Between Subjects Effects | | | | | | | | | | | | | |
| --- | --- | --- | --- | --- | --- | --- | --- | --- | --- | --- | --- | --- | --- |
|
|  | | Sum of Squares | | df | | Mean Square | | F | | p | | η²p | |
| Trial Order |  | 0.0576 |  | 1 |  | 0.0576 |  | 1.70 |  | 0.203 |  | 0.057 |  |
| Residual |  | 0.9480 |  | 28 |  | 0.0339 |  |  |  |  |  |  |  |
|  |  |  |  |  |  |  |  |  |  |  |  |  |  |
| --- | --- | --- | --- | --- | --- | --- | --- | --- | --- | --- | --- | --- | --- |
| Note. Type 3 Sums of Squares | | | | | | | | | | | | | |
|  | | | | | | | | | | | | | |
|  | | | | | | | | | | | | | |

## Estimated Marginal Means

### Trial Order ✻ ITI

#### 

[6]

# References

[1]
The jamovi project (2019). *jamovi*. (Version 1.1) [Computer Software]. Retrieved from https://www.jamovi.org.

[2]
R Core Team (2018). *R: A Language and envionment for statistical computing*. [Computer software]. Retrieved from https://cran.r-project.org/.

[3]
Morey, R. D., & Rouder, J. N. (2018). *BayesFactor: Computation of Bayes Factors for Common Designs*. [R package]. Retrieved from https://cran.r-project.org/package=BayesFactor.

[4]
Rouder, J. N., Speckman, P. L., Sun, D., Morey, R. D., & Iverson, G. (2009). Bayesian t tests for accepting and rejecting the null hypothesis. *Psychonomic Bulletin & Review, 16*, 225-237.

[5]
Singmann, H. (2018). *afex: Analysis of Factorial Experiments*. [R package]. Retrieved from https://cran.r-project.org/package=afex.

[6]
Lenth, R. (2018). *emmeans: Estimated Marginal Means, aka Least-Squares Means*. [R package]. Retrieved from https://cran.r-project.org/package=emmeans.
