# Supplementary material for: Temporal predictability does not impact attentional blink performance: effects of fixed vs. random inter-trial intervals
Source: PeerJ. 2020 Mar 5;8:e8677. doi: 10.7717/peerj.8677 (PMC7060903; doi:10.7717/peerj.8677)
Supplement: Supplemental Information 8 — This file should enable the reader to replicate all the statistical analyses in the paper using any statistical program [file peerj-08-8677-s008.csv › index.html]

Results


# Paired Samples T-Test

| Paired Samples T-Test | | | | | | | | | | | | | | | | | | | | | | | |
| --- | --- | --- | --- | --- | --- | --- | --- | --- | --- | --- | --- | --- | --- | --- | --- | --- | --- | --- | --- | --- | --- | --- | --- |
|  | | | | | | | | | | | | | | | | | | 95% Confidence Interval | | | |  | |
|  | |  | |  | | statistic | | ±% | | df | | p | | Mean difference | | SE difference | | Lower | | Upper | | Cohen's d | |
| T1 Accuracy percent fixed |  | T1 Accuracy percent random |  | Student's t |  | 0.643 |  |  |  | 29.000 |  | 0.525 |  | 1.556 |  | 2.421 |  | -3.395 |  | 6.507 |  | 0.117 |  |
|  |  |  |  | Bayes factor₁₀ |  | 0.235 |  | 4.002e-5 |  |  |  |  |  |  |  |  |  |  |  |  |  |  |  |
|  |  |  |  | Wilcoxon W |  | 104.500 | ᵃ |  |  |  | | 1.000 |  | -7.761e−5 |  | 2.421 |  | -4.165 |  | 5.000 |  | 0.117 |  |
| L8 Accuracy Fixed |  | L3 Accuracy Fixed |  | Student's t |  | 6.884 |  |  |  | 29.000 |  | < .001 |  | 0.182 |  | 0.026 |  | 0.128 |  | 0.236 |  | 1.257 |  |
|  |  |  |  | Bayes factor₁₀ |  | 105735.604 |  | 4.075e-10 |  |  |  |  |  |  |  |  |  |  |  |  |  |  |  |
|  |  |  |  | Wilcoxon W |  | 465.000 |  |  |  |  | | < .001 |  | 0.168 |  | 0.026 |  | 0.113 |  | 0.240 |  | 1.257 |  |
| L8 Accuracy Random |  | L3 Accuracy Random |  | Student's t |  | 5.593 |  |  |  | 29.000 |  | < .001 |  | 0.167 |  | 0.030 |  | 0.106 |  | 0.229 |  | 1.021 |  |
|  |  |  |  | Bayes factor₁₀ |  | 4073.721 |  | 8.315e-9 |  |  |  |  |  |  |  |  |  |  |  |  |  |  |  |
|  |  |  |  | Wilcoxon W |  | 446.000 |  |  |  |  | | < .001 |  | 0.148 |  | 0.030 |  | 0.103 |  | 0.212 |  | 1.021 |  |
| Fixed Interval Blink Magnitude |  | Random Interval Blink Magnitude |  | Student's t |  | 0.486 |  |  |  | 29.000 |  | 0.631 |  | 0.014 |  | 0.030 |  | -0.046 |  | 0.075 |  | 0.089 |  |
|  |  |  |  | Bayes factor₁₀ |  | 0.217 |  | 7.355e-5 |  |  |  |  |  |  |  |  |  |  |  |  |  |  |  |
|  |  |  |  | Wilcoxon W |  | 267.000 |  |  |  |  | | 0.490 |  | 0.022 |  | 0.030 |  | -0.038 |  | 0.075 |  | 0.089 |  |
|  |  |  |  |  |  |  |  |  |  |  |  |  |  |  |  |  |  |  |  |  |  |  |  |
| --- | --- | --- | --- | --- | --- | --- | --- | --- | --- | --- | --- | --- | --- | --- | --- | --- | --- | --- | --- | --- | --- | --- | --- |
| ᵃ 10 pair(s) of values were tied | | | | | | | | | | | | | | | | | | | | | | | |
|  | | | | | | | | | | | | | | | | | | | | | | | |
|  | | | | | | | | | | | | | | | | | | | | | | | |

| Test of Normality (Shapiro-Wilk) | | | | | | | | | |
| --- | --- | --- | --- | --- | --- | --- | --- | --- | --- |
|
|  | |  | |  | | W | | p | |
| T1 Accuracy percent fixed |  | - |  | T1 Accuracy percent random |  | 0.680 |  | < .001 |  |
| L8 Accuracy Fixed |  | - |  | L3 Accuracy Fixed |  | 0.909 |  | 0.014 |  |
| L8 Accuracy Random |  | - |  | L3 Accuracy Random |  | 0.915 |  | 0.020 |  |
| Fixed Interval Blink Magnitude |  | - |  | Random Interval Blink Magnitude |  | 0.974 |  | 0.657 |  |
|  |  |  |  |  |  |  |  |  |  |
| --- | --- | --- | --- | --- | --- | --- | --- | --- | --- |
| Note. A low p-value suggests a violation of the assumption of normality | | | | | | | | | |
|  | | | | | | | | | |
|  | | | | | | | | | |

| Descriptives | | | | | | | | | | | |
| --- | --- | --- | --- | --- | --- | --- | --- | --- | --- | --- | --- |
|
|  | | N | | Mean | | Median | | SD | | SE | |
| T1 Accuracy percent fixed |  | 30 |  | 88.166 |  | 90.835 |  | 7.783 |  | 1.421 |  |
| T1 Accuracy percent random |  | 30 |  | 86.610 |  | 89.165 |  | 10.443 |  | 1.907 |  |
| L8 Accuracy Fixed |  | 30 |  | 0.906 |  | 0.931 |  | 0.081 |  | 0.015 |  |
| L3 Accuracy Fixed |  | 30 |  | 0.724 |  | 0.755 |  | 0.165 |  | 0.030 |  |
| L8 Accuracy Random |  | 30 |  | 0.919 |  | 0.964 |  | 0.097 |  | 0.018 |  |
| L3 Accuracy Random |  | 30 |  | 0.752 |  | 0.762 |  | 0.174 |  | 0.032 |  |
| Fixed Interval Blink Magnitude |  | 30 |  | 0.182 |  | 0.142 |  | 0.145 |  | 0.026 |  |
| Random Interval Blink Magnitude |  | 30 |  | 0.167 |  | 0.164 |  | 0.164 |  | 0.030 |  |
|  |  |  |  |  |  |  |  |  |  |  |  |
| --- | --- | --- | --- | --- | --- | --- | --- | --- | --- | --- | --- |
|  | | | | | | | | | | | |
|  | | | | | | | | | | | |

## Plots

### T1 Accuracy percent fixed - T1 Accuracy percent random

#### 

#### 

### L8 Accuracy Fixed - L3 Accuracy Fixed

#### 

#### 

### L8 Accuracy Random - L3 Accuracy Random

#### 

#### 

### Fixed Interval Blink Magnitude - Random Interval Blink Magnitude

#### 

#### 

# Descriptives

| Descriptives | | | | | | | | | |
| --- | --- | --- | --- | --- | --- | --- | --- | --- | --- |
|
|  | | T1 Accuracy percent fixed | | T1 Accuracy percent random | | Fixed Interval Blink Magnitude | | Random Interval Blink Magnitude | |
| N |  | 30 |  | 30 |  | 30 |  | 30 |  |
| Missing |  | 0 |  | 0 |  | 0 |  | 0 |  |
| Mean |  | 88.166 |  | 86.610 |  | 0.182 |  | 0.167 |  |
| Median |  | 90.835 |  | 89.165 |  | 0.142 |  | 0.164 |  |
| Standard deviation |  | 7.783 |  | 10.443 |  | 0.145 |  | 0.164 |  |
| Variance |  | 60.577 |  | 109.065 |  | 0.021 |  | 0.027 |  |
| Minimum |  | 61.670 |  | 41.670 |  | 0.001 |  | -0.097 |  |
| Maximum |  | 100.000 |  | 93.330 |  | 0.512 |  | 0.608 |  |
|  |  |  |  |  |  |  |  |  |  |
| --- | --- | --- | --- | --- | --- | --- | --- | --- | --- |
|  | | | | | | | | | |
|  | | | | | | | | | |

# TOST Paired Samples T-Test

| TOST Results | | | | | | | | | | | |
| --- | --- | --- | --- | --- | --- | --- | --- | --- | --- | --- | --- |
|
|  | |  | |  | | t | | df | | p | |
| Fixed Interval Blink Magnitude |  | Random Interval Blink Magnitude |  | t-test |  | 0.486 |  | 29 |  | 0.631 |  |
|  | |  | | TOST Upper |  | -1.431 |  | 29 |  | 0.082 |  |
|  | |  | | TOST Lower |  | 2.403 |  | 29 |  | 0.011 |  |
| T1 Accuracy percent fixed |  | T1 Accuracy percent random |  | t-test |  | 0.643 |  | 29 |  | 0.525 |  |
|  | |  | | TOST Upper |  | -1.274 |  | 29 |  | 0.106 |  |
|  | |  | | TOST Lower |  | 2.560 |  | 29 |  | 0.008 |  |
|  |  |  |  |  |  |  |  |  |  |  |  |
| --- | --- | --- | --- | --- | --- | --- | --- | --- | --- | --- | --- |
|  | | | | | | | | | | | |
|  | | | | | | | | | | | |

| Equivalence Bounds | | | | | | | | | | | | | |
| --- | --- | --- | --- | --- | --- | --- | --- | --- | --- | --- | --- | --- | --- |
|  | | | | | | | | | | 90% Confidence interval | | | |
|  | |  | |  | | Low | | High | | Lower | | Upper | |
| Fixed Interval Blink Magnitude |  | Random Interval Blink Magnitude |  | Cohen's d |  | -0.350 |  | 0.350 |  |  |  |  |  |
|  | |  | | Raw |  | -0.057 |  | 0.057 |  | -0.036 |  | 0.065 |  |
| T1 Accuracy percent fixed |  | T1 Accuracy percent random |  | Cohen's d |  | -0.350 |  | 0.350 |  |  |  |  |  |
|  | |  | | Raw |  | -4.641 |  | 4.641 |  | -2.557 |  | 5.669 |  |
|  |  |  |  |  |  |  |  |  |  |  |  |  |  |
| --- | --- | --- | --- | --- | --- | --- | --- | --- | --- | --- | --- | --- | --- |
|  | | | | | | | | | | | | | |
|  | | | | | | | | | | | | | |

| Descriptives | | | | | | | | | | | |
| --- | --- | --- | --- | --- | --- | --- | --- | --- | --- | --- | --- |
|
|  | | N | | Mean | | Median | | SD | | SE | |
| Fixed Interval Blink Magnitude |  | 30 |  | 0.182 |  | 0.142 |  | 0.145 |  | 0.026 |  |
| Random Interval Blink Magnitude |  | 30 |  | 0.167 |  | 0.164 |  | 0.164 |  | 0.030 |  |
| T1 Accuracy percent fixed |  | 30 |  | 88.166 |  | 90.835 |  | 7.783 |  | 1.421 |  |
| T1 Accuracy percent random |  | 30 |  | 86.610 |  | 89.165 |  | 10.443 |  | 1.907 |  |
|  |  |  |  |  |  |  |  |  |  |  |  |
| --- | --- | --- | --- | --- | --- | --- | --- | --- | --- | --- | --- |
|  | | | | | | | | | | | |
|  | | | | | | | | | | | |

## Plots

### Fixed Interval Blink Magnitude - Random Interval Blink Magnitude

### T1 Accuracy percent fixed - T1 Accuracy percent random

# References

[1]
The jamovi project (2019). *jamovi*. (Version 1.0) [Computer Software]. Retrieved from https://www.jamovi.org.

[2]
R Core Team (2018). *R: A Language and envionment for statistical computing*. [Computer software]. Retrieved from https://cran.r-project.org/.
